# The Mycobacterium Tuberculosis FAS-II Dehydratases and Methyltransferases Define the Specificity of the Mycolic Acid Elongation Complexes

**DOI:** 10.1371/journal.pone.0029564

**Published:** 2011-12-22

**Authors:** Sylvain Cantaloube, Romain Veyron-Churlet, Nabila Haddache, Mamadou Daffé, Didier Zerbib

**Affiliations:** 1 Centre National de la Recherche Scientifique (CNRS), Institut de Pharmacologie et de Biologie Structurale (IPBS), Toulouse, France; 2 Université de Toulouse; Université Paul Sabatier (UPS), Toulouse, France; Fundació Institut d’Investigació en Ciències de la Salut Germans Trias i Pujol - Universitat Autònoma de Barcelona - CIBERES, Spain

## Abstract

**Background:**

The human pathogen *Mycobacterium tuberculosis* (*Mtb*) has the originality of possessing a multifunctional mega-enzyme FAS-I (Fatty Acid Synthase-I), together with a multi-protein FAS-II system, to carry out the biosynthesis of common and of specific long chain fatty acids: the mycolic acids (MA). MA are the main constituents of the external mycomembrane that represents a tight permeability barrier involved in the pathogenicity of *Mtb*. The MA biosynthesis pathway is essential and contains targets for efficient antibiotics. We have demonstrated previously that proteins of FAS-II interact specifically to form specialized and interconnected complexes. This finding suggested that the organization of FAS-II resemble to the architecture of multifunctional mega-enzyme like the mammalian mFAS-I, which is devoted to the fatty acid biosynthesis.

**Principal Findings:**

Based on conventional and reliable studies using yeast-two hybrid, yeast-three-hybrid and *in vitro* Co-immunoprecipitation, we completed here the analysis of the composition and architecture of the interactome between the known components of the *Mtb* FAS-II complexes. We showed that the recently identified dehydratases HadAB and HadBC are part of the FAS-II elongation complexes and may represent a specific link between the core of FAS-II and the condensing enzymes of the system. By testing four additional methyltransferases involved in the biosynthesis of mycolic acids, we demonstrated that they display specific interactions with each type of complexes suggesting their coordinated action during MA elongation.

**Significance:**

These results provide a global update of the architecture and organization of a FAS-II system. The FAS-II system of *Mtb* is organized in specialized interconnected complexes and the specificity of each elongation complex is given by preferential interactions between condensing enzymes and dehydratase heterodimers. This study will probably allow defining essential and specific interactions that correspond to promising targets for *Mtb* FAS-II inhibitors.

## Introduction

Multifunctional mega-enzymes such as fatty acid synthases (FAS) are comparable to enzymatic assembly lines [1], [2], [3]. FAS are responsible for the biosynthesis of fatty acids in all living organisms and are, schematically, of two types. A type-I FAS (FAS-I), found mainly in eukaryotes as a multifunctional enzyme with different degrees of homo- or hetero-multimerization, and a type-II FAS (FAS-II), more specific of prokaryotes and organelles, which is composed of monofunctional enzymes encoded by discrete genes. Several 3D structures of FAS enzymes are known and the structure of a mammal FAS (mFAS-I) has now been resolved at a high resolution [1], [4], [5], [6], [7]. According to these structures, mega-enzymes appear to have retained, or lost, functional domains in accordance with their biological functions but their overall structure, ultrastructure and the links between enzymatic modules are kept, with only minor changes.

**Figure 1:**
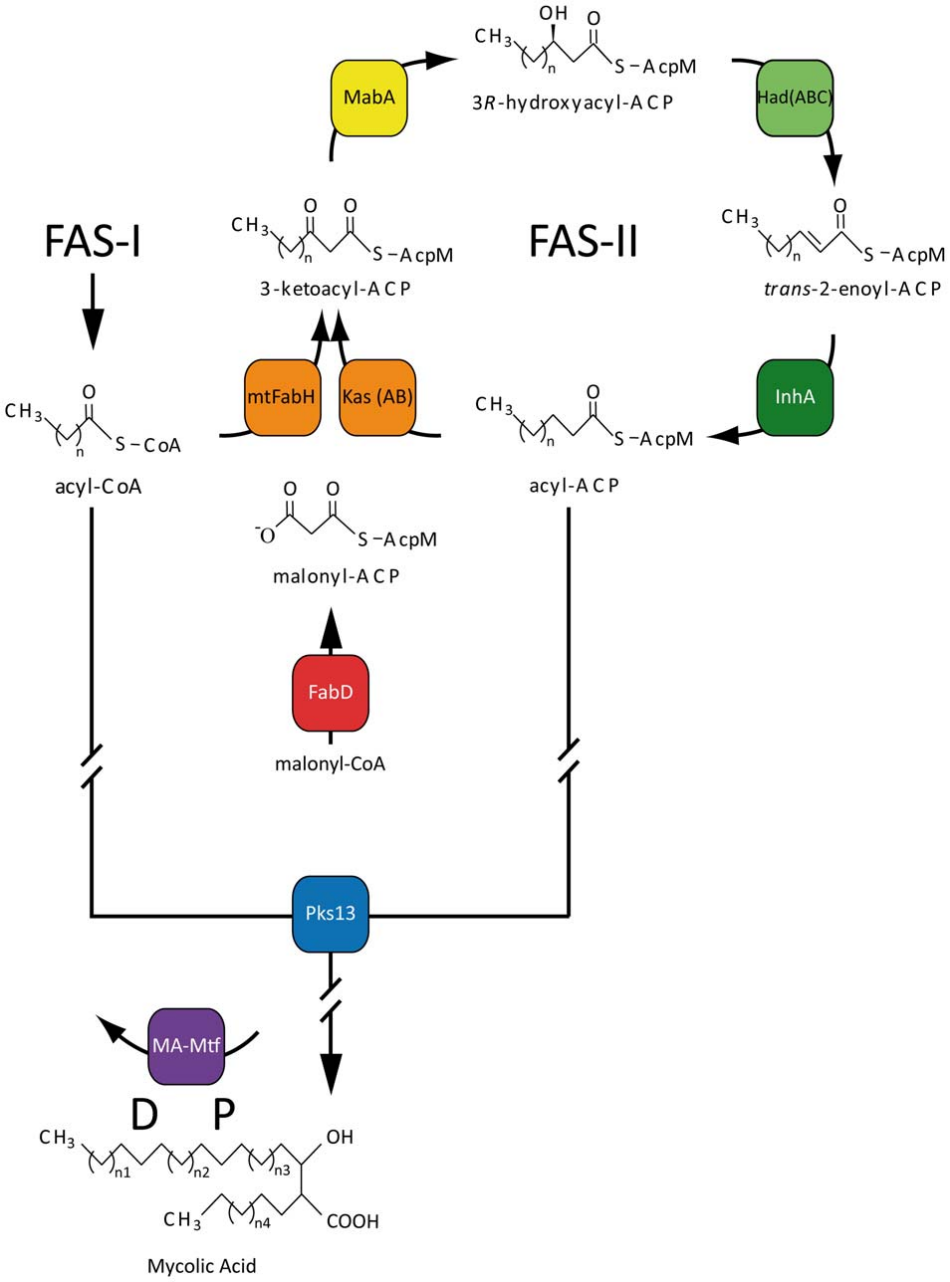
Schematic representation of the mycolic acid biosynthesis pathway. The biochemical reactions necessary to achieve the biosynthesis of mature mycolic acids are symbolized with black arrows. The enzymes responsible for each reaction are named in colored squares. The cofactors necessary for certain reactions to occur (NADPH, NADH) were omitted for clarity. The proximal and distal position of the meromycolic chain modifications by the MA-Mtfs are indicated as P and D respectively. The interrupted arrows are used to symbolize the existence of intermediates biochemical reactions that are not detailed on the figure.

The pathogenic bacillus *Mycobacterium tuberculosis* (*Mtb)* is the etiologic agent of tuberculosis which remains a major cause of death worldwide, and recently became even more worrying because of the emergence of multi-drug-resistant (MDR) and extensively-drug-resistant (XDR) clinical isolates [8]. *Mtb*, as the other mycobacteria, possesses a peculiar way of achieving the synthesis of fatty acids since a multifunctional, “eukaryotic like”, FAS-I and a prokaryotic "dissociated" FAS-II coexist in the bacteria [9]. These two systems, in *Mtb,* are devoted to the synthesis of normal chain-length fatty acids together with specific long-chain, α-branched and β-hydroxylated fatty acids: the mycolic acids (MA) [10]. MA represent the major and the most specific lipid components of the cell wall and are involved in what is now called the external myco-membrane of the Gram positive bacillus *Mtb* [11], [12], [13]. MA are part of the physiological barrier between the bacillus and its environment [14], [15]. The thick mycobacterium envelope is partly responsible for its inborn resistance to antibiotics and plays a major role in the virulence and the persistence of *Mtb*. The complexity, the essentiality and the variability of the biosynthetic pathway of MA (Fig. 1) probably reflect the fact that these fatty acids are of primary importance in the life of the bacillus. Several antituberculous agents, and specially isoniazid (INH), target the essential biosynthetic pathway of MA.

MA result from the Claisen condensation of an aliphatic chain of medium length (C_24_-C_26_) with a long mero-mycolic chain (up to C_60_) bearing specific biochemical modifications [16], [17]. The FAS-II initial substrates for the synthesis of the meromycolic chain are medium length keto-acyl-ACP resulting from the condensation by the mtFabH protein of the acyl-CoA products of FAS-I with malonyl-ACP produced by a malonylCoA ACP transacylase (MCAT) mtFabD [18]. After reduction by the keto-acyl-ACP reductase (KR) MabA [19], [20], [21], then dehydration by the recently identified hetero-dimers HadAB and HadBC hydroxyl-acyl-dehydratase (DH) [22], [23], [24] and finally the reduction by the enoyl-ACP reductase (ER) InhA [25], the fully saturated acyl-chain enter into a new cycle of elongation *via* the condensation by the keto synthase (KS) KasA or KasB with a new malonyl-ACP unit [26], [27], [28]. The meromycolic chains are modified at two specific positions: the distal (D) and proximal (P) positions (Fig. 1). To date, eight different methyltransferases (Mtf) have been involved in these specific modifications; there are MmaA1 to MmaA4, CmaA1, CmaA2, PcaA, and UmaA. After its synthesis the meromycolic chain is adenylated and ligated by FadD32 onto Pks13 [29], [30], [31], [32], [33], which is the terminal condensing enzyme that links the meromycolic chain to a carboxylated alpha chain coming from FAS-I. The remaining keto function of what will become the mycolic motif is then reduced, probably by the orthologous of *cmrA* (*rv2509*) identified in *Corynebacterium glutamicum* [34] and *Mycobacterium smegmatis* [35].

**Figure 2:**
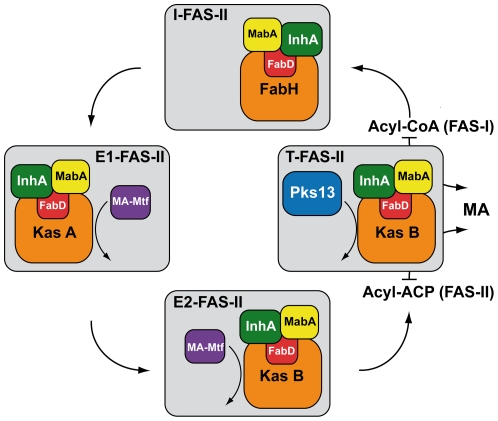
The FAS-II specialized complexes. The FAS-II initiation complex (I-FAS-II), elongation complex 1 and 2 (E1-FAS-II and E2-FAS-II), and the termination complex (T-FAS-II) are represented as filled grey boxes. The condensing enzymes KasA, KasB and MtFabH (labeled as FabH) are in orange. The reductases InhA and MabA are respectively represented in green and yellow. The malonylCoA ACP transacylase MtFabD is represented as a red box and labeled as FabD. The interactions between KasA and KasB with either the MA-Mtf (in violet) or the condensing enzyme Pks13 (in blue) are symbolized by curved arrows. The traffic of the substrates and products of each complex is also symbolized by curved arrows and the numbers represent the sequence of the events. MA is for mycolic acids, FAS-I for fatty acid synthase of type-I and FAS-II for FAS of type-II. The product of FAS-I and substrate of FAS-II is an Acyl-CoA. The product of FAS-II is an Acyl-ACP.

The essential interactions between the proteins of this biosynthetic pathway represent very promising targets *per se*. In order to approach this final objective, we have previously analyzed the complex network of interactions between the main FAS-II protein components. We have proposed the first model of the architecture of a prokaryotic FAS-II system [36], [37]. In this model of interactome, called here the MABI (**M**ycolic **A**cid **B**iosynthesis **I**nteractome), three types of FAS-II specialized complexes are interconnected together (Fig. 2): (i) the ‘initiation FAS-II’ (I-FAS-II) is formed by a core (the reductases and mtFabD) and mtFabH and represent the link between FAS-I and FAS-II; (ii) two ‘elongation FAS-II’ (E-FAS-II) complexes consist in a core and either KasA (E1-FAS-II) or KasB (E2-FAS-II) and are capable of elongating acyl-AcpM to produce full-length meromycolyl-AcpM ; and finally (iii) the ‘termination FAS-II’ (T-FAS-II) involves Pks13 linked with KasB and condenses the α-branch with the meromycolic branch. Our working hypothesis was that the specialized and interconnected complexes of the prokaryotic “dissociated” FAS-II system of *Mtb*, might adopt the same composition and architecture as a multifunctional FAS-I protein.

The aim of the present work was to obtain a precise and global view of the MABI of the FAS-II complexes of *Mtb* and address the question of the specificity of each specialized complex by including the recently identified dehydratases of the FAS-II system (HadAB and HadBC) and specific methyltransferases involved in meromycolic chain modifications in the interactome.

## Results

### The Had dehydratases form specific heterodimers in the Y2H system

Using a biochemical approaches together with a mycobacterial two-hybrid system, It has been shown in our laboratory that the dehydration step of FAS-II was accomplished by three proteins (HadA, HadB and HadC) [22], [24]. The heterodimerization of the dehydratase proteins conducted to the formation of two types of active heterodimers HadAB and HadBC. In order to be able to set up experiments using a yeast-three-hybrid (Y3H) system allowing to test the interaction of each heterodimer with a given FAS-II protein, we first analyzed the heterodimer formation in a Y2H screen. The *Mtb* genes *rv0635, rv0636*, *rv0637* coding respectively for HadA, HadB, and HadC were inserted into the Y2H vectors (pGAD-T7 and pGBK-T7) to produce in-phase C-terminal fusions with the coding sequences of either the activator domain (AD) or the binding domain (BD) of the yeast transcriptional activator GAL4. All the possible combinations of pGAD-T7 and pGBK-T7 derivatives were transformed in the yeast strain AH109. As negative controls, the interactions between the three Had proteins fused to either GAL4 domains were tested against the empty vectors (pGAD-T7 or pGBK-T7) or the lamin fusion from Clontech (pGBK::*lam*). The results obtained with the positive control pair (pGAD::*AgT* and the pGBK::*p53)* are not shown. The three proteins were tested in both directions, that is to say merged either to the activator domain (AD) or the binding domain (BD) of GAL4 and on each selective medium using the “two screen test” described in Materials and methods and before [36], [37]. Homotypic interactions were revealed for HadA and HadB but not for HadC (Table 1), a result in agreement with the previous findings indicating that the only relevant homodimers concerned HadA or HadB [22]. The specific formation of the HadAB and HadBC heterodimers was also clearly observed in this Y2H system whereas HadAC was never observed. The pertinence of the “both direction Y2H screen” was well illustrated by the result obtained in yeast with BD-HadB. In contrary to the AD-HadB fusion that interacted clearly with HadC or HadA as previously described, the BD-HadB fusion did not interact with either HadA or HadC. We excluded bellow the results obtained with the BD-HadB fusion that did not interact with any protein tested (data not shown).

**Table 1:** Y2H analysis of interactions between the Had monomers.

AD fusions	BD fusions			
	*lam*	*hadA*	*hadB*	*hadC*
Ø	-a	-	-	-
*hadA*	-	+/-	-	-
*hadB*	-	+	+/-	+
*hadC*	-	-	-	-

a Each sign indicates the growth on three selective media (DOBA-LTH, DOBA-LTA, and DOBA-LTHA) the rules of attribution of either +, +/-, or - are given in Materials and Methods.

### Each Had heterodimer contacts preferentially a specific FAS-II complex

The Y3H system is based on the reconstitution, in yeast, of a functional GAL4 activator with a protein bridge between the AD and the BD fusions proteins. The expression of the protein bridge was controlled by a pMet promoter (repressed by methionine) present on a derivative of pGBK-T7 (pBridge) that expresses constitutively a given BD fusion. Each pBridge derivative, containing one *had* gene expressed as a constitutive BD fusion and the other one as a methionine repressible gene, was used to test interactions with the FAS-II genes AD-fusions carried on a pGAD-T7 derivatives. The Y3H screen was essentially conducted like the Y2H screen, except that half of the selective plates contained methionine in order to repress the pMet promoter located on the pBridge constructs (Table 2). Each protein HadA or HadC did not interact individually with FAS-II protein (Table 2, columns 4 and 5, + Met). HadA and HadC, which do not interact with each other (Table 1), do not interact either with the FAS-II components when they are co-produced (Table 2, columns 4 and 5, - Met). In contrast, we found interactions with the condensing enzymes KasA, KasB, or mtFabH when the pairs HadAB or HadBC were tested (Table 2, columns 2 and 3). HadAB interacted with the three condensing enzymes with a preference for KasA and HadBC interacted with only KasB. The interaction seen in the presence of methionine (Column 2 and 3, + Met) might reflect a leak of the pMet promoter allowing the HadB production because HadA and HadC do not interact when they are alone (Table 2, columns 4 and 5, + Met). Each heterodimer HadAB or HadBC interacted with the condensing enzymes probably *via* an interaction through HadB. The specificity of interaction seemed to come from either HadA (KasA preference) or HadC (KasB preference) when they were in the heterodimer. HadAB might contact I-FAS-II and E1-FAS-II whereas HadBC might interact with only E2-FAS-II.

**Table 2:** Y3H analysis of protein-protein interactions between the Had heterodimers and the FAS-II proteins.

AD fusions	BD gene fusions and *pmet* operon fusions								
	1	2		3		4		5	
	BD-lam	BD-HadA		BD-HadC		BD-HadC		BD-HadA	
		*pmet*-HadB		*pmet*-HadB		*pmet*-HadA		*pmet*-HadC	
		Met^a^		Met		Met		Met	
Ø	-b	-	-	-	-	-	-	-	-
*mabA*	-	-	-	-	-	-	-	-	-
*inhA*	-	-	-	-	-	-	-	-	-
*kasA*	-	+/-	+	-	-	-	-	-	-
*kasB*	-	-	+/-	+/-	+/-	-	-	-	-
*mtfabH*	-	-	+/-	-	-	-	-	-	-
*mtfabD*	-	-	-	-	-	-	-	-	-

a Met Indicates the presence of 1mM methionine in the medium.

b Each sign symbolizes the growth on three selective media (DOBA-LTH, DOBA-LTA, and DOBA-LTHA).The rules of attribution of either +, +/-, or - are given in Materials and Methods.

### The Mycolic acid-Mtfs (MA-Mtf) interact with the Elongation Complexes

We have previously demonstrated that the four MA-Mtfs MmaA1 to MmaA4, involved in meromycolic acid modifications, interacted mainly with the condensing enzymes KasA and KasB and not with mtFabH [36], [37]. MA-Mtfs were shown to be specific for the elongation complexes E1-FAS-II and E2-FAS-II [36], [37]. We investigated here the interactions between the FAS-II proteins and four additional MA-Mtfs involved in the meromycolic chain modification: CmaA1, CmaA2, PcaA, and UmaA. We used *in vivo* (Y2H) and *in vitro* (Co-IP) complementary experimental approaches. The *Mtb* genes *rv3392c*, *rv2254c, rv0470c*, *rv0469* coding respectively for CmaA1, CmaA2, PcaA, and UmaA were inserted into the Y2H vectors (pGAD-T7 and pGBK-T7). The pGAD-T7 and pGBK-T7 derivatives expressing the fusions of the four MA-Mtfs genes *mmaA1* to *mmaA4* and of the main FAS-II proteins were already had available from a previous study [36], [37]. All the possible combinations of double transformants of the yeast strain AH109 were tested in Y2H with the suitable positive and negative controls. We analyzed the results obtained when the proteins were tested in “both direction” (Table S1) and found, according to their behavior with respect to the MA-Mtf proteins three groups of FAS-II proteins. In Y2H, the reductases InhA and MabA, did not interact with any MA-Mtfs. In contrast, KasA, KasB and mtFabD interacted with nearly all the MA-Mtfs with some variations in the strength of the interactions. Finally the condensing enzymes mtFabH and Pks13, either did not interact or interacted only poorly with some MA-Mtf.

### The MA-Mtfs contacts specifically each type of FAS-II complex

**Figure 3:**
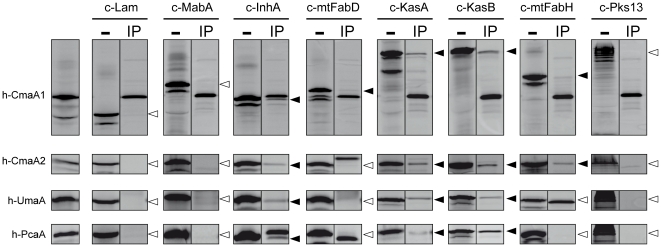
*In vitro* Co-IP between *Mtb* FAS-II proteins and MA-Mtfs. The L-[^35^S]-methionine labeled h-proteins (HA tagged; h-protein) and c-proteins (c-myc tagged; c-protein) from *in vitro* transcription translation reactions and Co-IP reactions products were fractionated by SDS-PAGE (10%) followed by phosphor-imaging analysis. In column 1, the gels contained the h-protein alone. In even numbered column, the gels contained the c-protein alone. In the other columns (odd numbered except 1), the gel contained the Co-IP reaction products. Each row corresponds to a distinct h-protein analysis and the names of proteins are indicated in front of their position of migration. For CmaA1, the complete gel lines are presented. For the other Mtfs only the gel region of interest is presented. The black filled arrows represent a Co-IP band corresponding to a positive interaction. The open arrows mark the position of the absent Co-IP band and correspond to a negative interaction.

The pGAD-T7 and pGBK-T7 plasmids are organized in such a way that they allow the *in vitro* synthesis of ^35^S labeled proteins tagged in N-terminus by either a c-Myc epitope (giving a c-protein from pGBK-T7 derivatives) or a HA epitope (giving a h-protein from pGAD-T7 derivatives). Co-immunoprecipitation (Co-IP) experiments were designed to analyze the *in vitro* interactions between the four MA-Mtfs and the FAS-II proteins by trapping c-proteins with h-protein bound onto magnetic beads coated with anti-HA antibodies (Fig. 3). *In vitro,* KasA and KasB behaved like in Y2H experiments and interacted with all the MA-Mtfs. It was less obvious for MtFabD that displayed only light interactions with only two MA-Mtfs (CmaA1 and CmaA2). MtFabH remains poorly interactive and displayed only faint interactions with CmaA1, CmaA2, and UmaA. Pks13 did not interact at all *in vitro*. Finally, and in contrast to the Y2H results, InhA interact very significantly with the four MA-Mtfs. We have observed this behavior previously with InhA and MabA [36], [37] that never displayed any interaction in Y2H but were interactive *in vitro*. We have attributed this behavior to the high degree of multimerization of these proteins [20], [38], [39] that might interfere in yeast with the reconstitution of an active GAL4. This was particularly true with MabA that interacted only if its multimerization was impaired by punctual mutations [37].

The compilation of the results of the two Y2H analyses (Table S1) together with those of the Co-IP analysis (Fig. 3 and Table S1) was obtained by giving arbitrarily the same weight to each experiment (Materials and Methods) to give a semi-quantitative view of the data (Table 3). We have defined three interaction groups. MabA felt in the non-interacting (NI) group whereas the other reductase, InhA, was found in the all interacting (AI) group because of the Co-IP results (Fig. 3). The condensing enzymes KasA and KasB were also part of the AI group together with MtFabD. Finally, Pks13 and MtFabH, because of either the absence of interaction with some of the MA-Mtfs tested or the weakness of interactions found, were placed in the poorly interacting (PI) group.

**Table 3:** Protein-protein interactions between MA-Mtf and FAS-II: a compilation of Y2H and Co-IP.

AD fusions	BD fusion									Group
	*lam*	*cmaA1*	*cmaA2*	*umaA*	*pcaA*	*mmaA1*	*mmaA2*	*mmaA3*	*mmaA4*	
Ø	-a	-	-	-	-	-	-	-	-	
*mabA*	-	-	-	-	-	-	-	-	-	NI^b^
*inhA*	-	+/-	+/-	+/-	+/-	+/-	+/-	+/-	+/-	AI
*kasA*	-	+	+	+	+	+/-	+	+	+	AI
*kasB*	-	+	+	+	+	+	+	+	+	AI
*mtfabH*	-	+/-	+/-	+/-	-	+/-	+/-	+/-	+/-	PI
*mtfabD*	-	+	+	+	+	+	+	+/-	+	AI
*pks13*	-	-	-	-	-	-	-	+/-	+/-	PI

a Each sign represent the compilation of the results of three experiments: the Y2H results in “both directions” (Table S1) and the Co-IP (Table S1 and Fig. 3). These three types of experiments were each scored as +, +/- or - as indicated in the text and in Materials and Methods.

b The interaction groups are indicated as defined in the text: AI (All Interacting Group), NI (Non Interacting Group) and PI (Poorly interacting Group).

**Figure 4:**
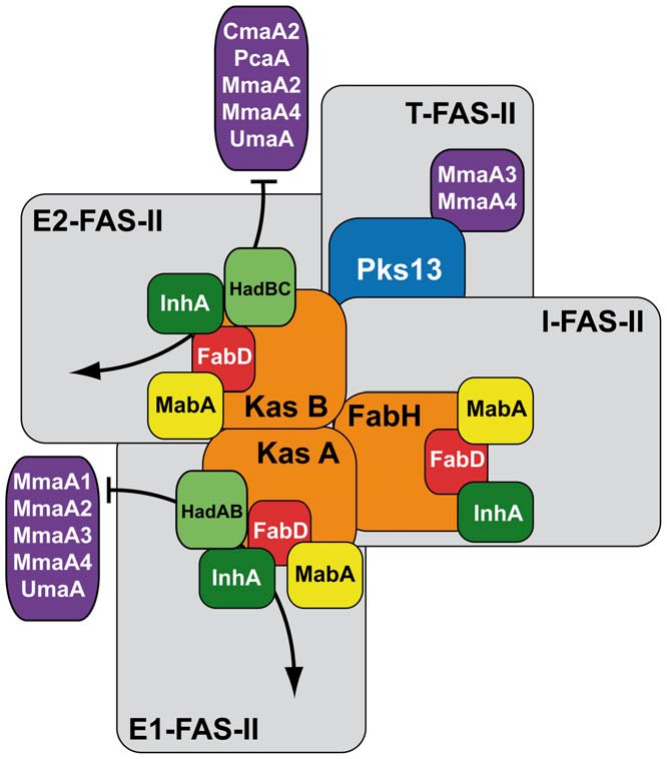
Summary of protein-protein interactions within the MA biosynthesis interactome. The protein-protein interactions between the components of the specialized interconnected FAS-II complexes of *Mtb* are represented as defined in the present work and before [36], [37]. The condensing enzymes (in orange) interact with each other and with the core composed of MabA (in yellow),InhA (in dark green) and FabD (in red). For clarity, the core is depicted in each type of complex. KasA and KasB interact preferentially with HadAB (in light green) and HadBC respectively. The interactions between the MA-Mtfs (in violet) and KasA, HadAB, FabD, and InhA, or KasB, HadBC InhA, and FabD are represented with black curved arrows. Pks13 (in blue) interact with KasB and with the MA-Mtfs MmaA3 and MmaA4. Each type of complex is represented as a grey rectangle; I-FAS-II is the initiation complex, E1-FAS-II and E2-FAS-II the elongation complexes of type 1 and 2 respectively and T-FAS-II is the termination complex.

The overall interaction pattern of the MA-Mtfs was clear and followed the classification of FAS-II complexes as I-FAS-II, E-FAS-II and T-FAS-II (Fig. 4). All the MA-Mtfs interacted preferentially with the condensing enzymes, KasA and KasB, and with mtFabD, suggesting a preference for the elongation complexes E1-FAS-II and E2-FAS-II. The MA-Mtfs of FAS-II might contact the E-FAS-II complexes mainly through interactions with the KS-AT proteins (KasA-KasB and mtFabD) and seemed to contact InhA more than MabA in the core of the FAS-II complexes.

### The Had dehydratases participate in the FAS-II complex specificity

We used the Y3H system to monitor the interactions between the Had heterodimers and all the MA-Mtf proteins. The pBridge derivatives expressing each heterodimer of Had were introduced in AH109 together with a pGAD-T7 derivative encoding a GAL4 AD fusion with a given MA-Mtf. For clarity, and because of the detrimental effect of the BD-HadB fusion (Table 1), the data obtained with the BD-HadB fusion with the pBridge BD-HadB pmet-HadA or the pBridge BD-HadB pmet-HadC were omitted from Table 4. All the results were compared with the negative controls as above (Table 4, line 1 and column 1). Five MA-Mtfs (CmaA2, MmaA1, MmaA3, CmaA1, and PcaA) displayed a clear preference for either of the two dehydratases. The CmaA2 protein interacted with HadBC when HadB was induced (Table 4, columns 3). In addition, CmaA2 did not interact with HadA or HadC alone (Table 4, columns 4 and 5). MmaA1 interacted with HadAB even when HadB was repressed (Table 4, column 2) this interaction with HadA alone was not seen when a putative background of HadC was present (Table 4, column 5). MmaA3 interacted with HadAB even in the absence of HadB (Table 4, column 2) and this interaction with HadA seemed inhibited by the presence of HadC (Table 4, column 5). The behavior of CmaA1 was not clear because it interacted only with HadA in only one type of double transformant (Table 4, column 2) and not in the other (Table 4, column 5). PcaA interacted with HadA (Table 4, column 2) and HadC (Table 4, columns 3 and 4). The interaction with HadA was inhibited by HadB (Table 4, column 2) and the interaction with HadC was not inhibited by either HadB or HadA (Table 4, columns 3 and 4). We concluded that PcaA interacted with HadBC through HadC. The three remaining MA-Mtfs (MmaA2, MmaA4, and UmaA) were more interactive. MmaA2 interacted with HadA (Table 4, columns 2 and 5) and HadC (Table 4, columns 3 and 4). These interactions were not inhibited by the presence of either Had proteins when the pMet promoters were not repressed (Table 4, columns 2 to 4) suggesting that MmaA2 was able to interact with both types of Had heterodimers (HadAB and hadBC). The MmaA4 protein (also named Hma [40]) displayed an interaction profile similar to the one of MmaA2 suggesting an interaction with both types of Had heterodimers. Finally, UmaA interacted with both type of Had heterodimers with no clear preference (Table 4, columns 2 to 4). To conclude, we observed a clear selectivity of the MA-Mtfs for each dehydratase heterodimer. MmaA2, MmaA4, and UmaA interacted with both Had heterodimers with no clear preference. In contrast MmaA1 and MmaA3 displayed a clear specificity for HadAB whereas CmaA2 and PcaA interacted preferentially with HadBC. It seems likely that Had heterodimers might represent a specificity determinant of the FAS-II complexes.

**Table 4:** Y3H analysis of protein-protein interactions between the Had heterodimers and the MA-Mtfs.

AD fusions	BD gene fusions and *pmet* operon fusions								
	1	2		3		4		5	
	BD-lam	BD-HadA		BD-HadC		BD-HadC		BD-HadA	
		*pmet*-HadB		*pmet*-HadB		*pmet*-HadA		*pmet*-HadC	
		Met		Met		Met		Met	
Ø	-a	-	-	-	-	-	-	-	-
*cmaA2*	-	-	-	-	+/-	-	-	-	-
*mmaA1*	-	+/-	+	-	-	-	-	-	-
*mmaA3*	-	+/-	+/-	-	-	-	-	+/-	-
*cmaA1*	-	+/-	-	-	-	-	-	-	-
*pcaA*	-	+/-	-	+/-	+/-	+	+	-	-
*mmaA2*	-	+	+	+/-	+	+	+	+/-	+/-
*mmaA4*	-	+/-	+/-	+/-	+/-	+/-	+/-	+	+
*UmaA*	-	+	+	+	+/-	+/-	+/-	+	+/-

a Each sign symbolize the growth on three selective media (DOBA-LTH, DOBA-LTA, and DOBA-LTHA).The rules of attribution of either +, +/-, or - are given in Materials and Methods. Met. Indicate the presence of 1mM methionine in the medium.

## Discussion

Our goal was first to achieve the characterization of the *Mtb* MABI by including interaction results obtained with the recently identified dehydratases HadABC of FAS-II together with MA-Mtfs involved in the modification of the meromycolic chains. Because these two types of protein are each specific for a given type of meromycolic chain, we also wanted to discover the potential molecular basis of the specificity of each already identified [36], [37] specialized FAS-II complex: I-FAS-II, E1-FAS-II, E2-FAS-II and T-FAS-II (Fig. 2).

We confirmed the formation of the specific heterodimers of dehydratases HadAB and HadBC in Y2H. These (3R)-hydroxyacyl-ACP dehydratases have been identified as responsible for the dehydration of the meromycolic chain during elongation by the FAS-II complexes [22], [24]. The HadB monomer is believed to carry the catalytic center [22]. Y3H experiment showed that the dehydatases interact with the FAS-II proteins through the HadB protein reinforcing the idea that they might belong to the FAS-II specialized complexes. The dehydratases interacted essentially with the condensing enzymes and we did not observe any interaction with the reductases MabA or InhA in Y3H. Rv3389c, a related *Mtb* dehydratase: that do not participate in MA biosynthesis [23] did not interact with any FAS-II protein (data not shown).Interestingly, we also established a selectivity of each heterodimer HadAB or HadBC for either KasA-mtFabH or KasB respectively. HadAB has been proposed to be involved, like KasA [41], [42], in the early elongation cycle of FAS-II of mycobacteria and in the synthesis of intermediate length fatty acids in Nocardia and Rhodococcus [22]. HadAB, which interacts with KasA, can be placed into the E1-FAS-II complex centered on KasA (Fig. 4). HadBC, like KasB [41], [42], was strongly suspected to be implicated in the late steps of the meromycolic chain biosynthesis. Indeed, HadBC prefers longer substrates than HadAB and there is neither HadC nor KasB orthologs in genera bearing medium-chain MA [22]. Our results showing a HadBC-KasB interaction are fully consistent with these observations and place HadBC into the E2-FAS-II complexes (Fig. 4). It is not possible yet to ascertain the existence of a physical link between the Had heterodimers and the reductases in the FAS-II complexes but each type of Had heterodimer likely belong specifically to each type of elongation complex *via* interactions with the condensing enzymes (Fig. 4). We have tried to confirm these results with Co-IP experiments *in vitro* (data not shown). However, each individual monomer of Had interacted with all the proteins tested, including with the controls. The same results were obtained when the Hads monomers were tested two-by-two against the FAS-II proteins (data not shown). We concluded that isolated monomers do not form proper heterodimers *in vitro* and that they probably expose hydrophobic surfaces which are responsible for the observed non-specific interactions. *In vivo*, the three *had* genes are nearly overlapping in a compact operon and we believe that the Had subunits of each heterodimer have probably to be produced in a coordinated fashion.

We have shown previously that the MA-Mtfs MmaA1 to MmaA4 can interact with the elongation complexes E-FAS-II through specific interactions with the keto-synthases KasA and KasB. Here, we showed that the four remaining MA-Mtfs (PcaA, UmaA, CmaA1, and CmaA2) behaved in the same way and interacted also with the FAS-II condensing enzymes. The FAS-II elongation complexes might possess at least one MA-Mtf binding site (Fig. 4). We propose that modifications of the meromycolic chain could occur during the elongation process in the FAS-II elongation complexes. The MA-Mtfs of mycobacteria modify the meromycolic chains of the MA at two specific positions referred as distal (D) and proximal (P) positions with respect to the mycolic motif (Fig. 1). During fatty acid biosynthesis, as described in text books, the meromycolic chain elongates from the carboxyl end. The D region is synthesized at the early step of elongation before the P region that is synthesized during the last steps of elongation. We have postulated that E1-FAS-II and E2-FAS-II could synthetize the portion of the meromycolic chain containing the D region and the P region respectively [36] and it has been shown that KasB is involved in the synthesis of the proximal portion of the mero mycolic chain in *M.marinum* [42] and in *Mtb* [41].

This hypothesis has been integrated in a regulation model of the functioning of FAS-II [43]. We have interpreted our data in the view of these models keeping in mind that the modifications of the meromycolic chains by MA-Mtfs might take place during the elongation process, as suggested by the effect on the proximal modification of the meromycolic chain of the disruption of KasB in *Mtb* [41]. A distal modification of the meromycolic chain by a MA-Mtf is supposed to occur in the E1-FAS-II complex before its proximal modification with another MA-Mtf in the E2-FAS-II complex. We showed that CmaA2 and PcaA interact preferentially with the HadBC heterodimers. It corresponds to an interaction with the E2-FAS-II complex centred on KasB and devoted to the late elongation steps. Both PcaA and CmaA2 modify the proximal position of the meromycolic chain by respectively *cis-* and *trans-* cyclopropanation of α-MA or oxygenated-MA [44], [45]. PcaA and CmaA2 must actually act in the last stages of the elongation process in the E2-FAS-II complexes. Conversely, MmaA3, which catalyzes the formation of the methoxy groups at the distal position of oxygenated-MA [46], [47] displayed a preference for HadA in the HadAB heterodimer. We put MmaA3 in the E1-FAS-II complex centered on KasA and involved in the early steps of elongation allowing methylation at the first modified position: the distal position. The profile of interaction of MmaA2 was also consistent with its known activities. MmaA2 has been involved in the *cis*-cyclopropanation of the distal position of α-MA and of the proximal position of oxygenated-MA [41], [48]. MmaA2 displayed a redundant function with CmaA2 for the *cis*-cyclopropanation of oxygenated MA [49]. MmaA2 interact with both types of Had heterodimers, suggesting that it can indeed participate in distal or proximal modification in either E1-FAS-I or E2-FAS-II complexes. The MA-Mtf CmaA1 has been initially suspected to synthetize a *cis*-cyclopropan at the distal position of α-MA when it was overproduced in *M.smegmatis* [50], [51]. This hypothesis has not been confirmed when it has been re-evaluated in *Mtb* KO-mutant studies [48]. The effective role of CmaA1 still remains unclear. Here, we observed a unique and light interaction between CmaA1 and HadA without any clear specificity for one or the other of the Had heterodimers. It suggests that CmaA1 is not part of the MABI. It is possible that when CmaA1 has been overproduced *in vivo* [50], the CmaA1-HadA interaction was sufficient to promote an artifactual targeting of CmaA1 to the E1-FAS-II complex in order to modify the D position of the meromycolic chain. The case of UmaA is different in the sense that even if it has been shown that UmaA methylates the vicinal position of proximal double bonds in α-MA and epoxy-MA of *M.smegmatis*, the *Mtb*-KO mutant had no phenotype [52]. Here, by looking at interactions between *Mtb* proteins, we observed UmaA interactions with both types of Had heterodimers. It suggests that UmaA participates in both types of FAS-II elongation complexes whereas it is supposed to act only at the P position [52] and to display a preference for the HadBC dimer of the E2-FAS-II complexes. This discrepancy between these results and the activity of UmaA in *M.smegmatis* could be explained by the fact that we studied only *Mtb* proteins and not *M.smegmatis* proteins and suggest that UmaA might have a function in *Mtb* that need to be identified. The only example, presented here, which was clearly not coherent with the literature, was the one of MmaA1. MmaA1 has been involved in the methylation of the vicinal position of the *trans*-cyclopropane in the P position of oxygenated-MA [53] and “should” prefer the HadBC dimer in the E2-FAS-II system. We observed the opposite result and a very clear specificity of interaction for HadAB in E1-FAS-II. It suggests an activity at the D position of the meromycolic chain. We believe that overproduction experiments [53] might conduct to a misleading interpretation of the MmaA1 activity like as demonstrated in the case of CmaA1 [48], [50], [51]. The precise definition of the role of MmaA1 awaits the study of a *Mtb* KO-mutant. Finally, MmaA4 (also named Hma [40]), is involved in the methylation of the vicinal position of the oxygenated motives in the distal region of the oxygenated-MA. Hma probably provide the substrates to MmaA3 for the synthesis of methoxy-MA and keto-MA [46], [54]. Hma was suspected to act on a double bond present at the distal position of proximal *cis*-cyclopropanated MA [55]. We observed a Hma-HadAB interaction that could explain its “distal” site of activity when belonging to the E1-FAS-II complex. In addition to the absence of oxygenated-MA in a *Mtb hma* KO-mutant, the MA chains are shortened, suggesting the untimely end of the elongation process and a functional interaction with the last stages of elongation [55], [56]. Furthermore, there is no *trans*-cyclopropanated MA (the product of CmaA2) in the *hma* mutant strain [55]. And finally, the deletion of the *kasB* gene in *Mtb* also provokes the disappearance of the *trans*-cyclopropanated MA, suggesting a CmaA2-KasB interaction [41]. It appears that the activities of Hma, KasB, and CmaA2 seem to be interdependent. This functional link between Hma and other proteins belonging to the E2-FAS-II complexes might explain the Hma-HadBC interaction observed here.

### The global organization of the FAS-II MABI

**Figure 5:**
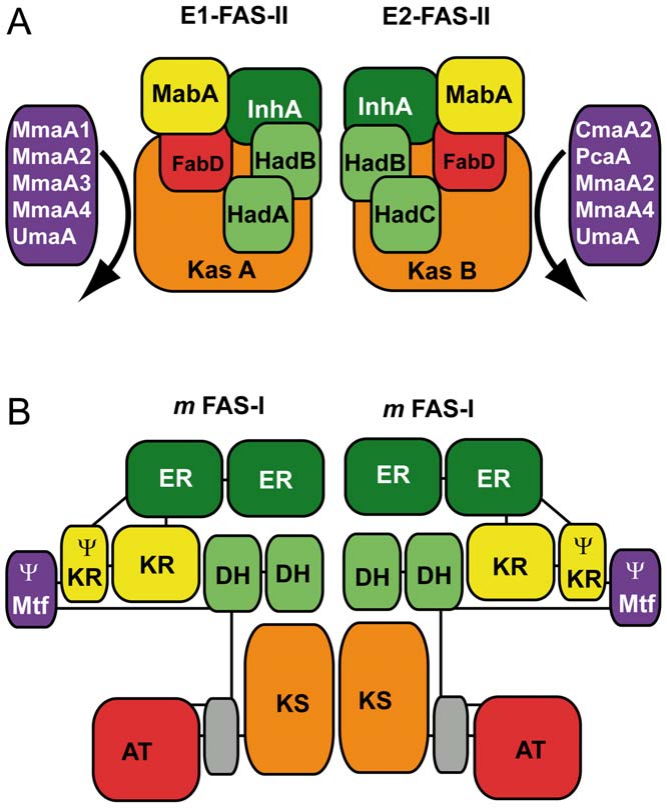
Modular organization of *Mtb* FAS-II and mFAS-I. (A) Schematic representation of type-I (E1-FAS-II) and type-II (E2-FAS-II) FAS-II elongation complexes as defined by the analysis of protein interactions in the present work and before [36], [37]. The interactions between the different MA-Mtfs (in violet) and each complex are represented by curved arrows. (B) Schematic representation of a dimer of m-FAS-I adapted from Maier and colleagues [6] and drawn from the 3D structure. For both panels, the enoyl reductase domains (ER) and proteins (InhA) are in dark green, the keto-reductase domains (KR) or pseudo keto-reductase domains (Ψ-KR) and proteins (MabA) are in yellow, the keto-synthase domains (KS) or proteins (KasA, KasB) are in orange, the acyl transferase domains or the MtFabD protein (FabD) are in red, the pseudo-methyltransferase domains (Ψ-Mtf) or the MA-Mtf proteins (CmaA1, CmaA2, MmaA1 to MmaA4, UmaA, PcaA) are in violet, the dehydratase domains (DH) or proteins (HadA, HadB, HadC) are in light green and the mammalian FAS-I linker region (L) are in grey. The links between the domains of mFAS-I in its primary structure are symbolized by straight lines.

The 3D structures of several FAS enzymes are known and the structure of a mammal FAS has now been resolved at high resolution [4], [5], [6], [7]. Even if the present study do not allow any structural comparison between the “dissociated” FAS-II and the megaenzyme mFAS-I, each type of FAS-II complex (Fig. 5A) can be compared, in term of its composition, to a monomer of the mFAS-I protein [6], [9], [36], [37].By completing this MABI we showed that it might adopt the same composition as a multifunctional eukaryotic FAS-I protein. The observed preferential Had-Kas interactions can be connected with the existence, in mFAS-I, of a KS-DH interaction [6]. HadAB or HadBC might select the keto-synthase needed for the elongation in a given FAS-II complex. It will be interesting to try to define the interaction interfaces between the Had and the Kas proteins. Interestingly, in mFAS-I, there is a pseudo-Mtf domain (Ψ-Mtf), consisting in a S-adenosyl-methionine binding site [6] facing the KR region of the protein (Fig. 5B). We discovered here specific interactions between the MA-Mtfs and FAS-II. The known structures of the MA-Mtfs are very similar [40], [57], [58] and it is thus possible to imagine that each MA-Mtf can contact FAS-II at a unique position and in the same manner. The prokaryotic “dissociated” FAS-II system of *Mycobacterium tuberculosis* (*Mtb*) might adopt the same architecture as a multifunctional eukaryotic FAS-I enzyme. We are actually investigating this possibility by using molecular modeling and structural alignments.

In addition to provide the first global view of the known components of the FAS-II mycolic acid biosynthesis interactome we identified its specificity determinants. The Had dimers, together with the keto-synthases KasA and KasB might target specifically the MA-Mtfs to a given elongation complex to perform either a distal modification (HadAB/KasA/E1-FAS-II) or a proximal modification (HadBC/KasB/E2-FAS-II) during the course of the meromycolic chain elongation. The FAS-II system of *Mycobacterium tuberculosis* is organized in specialized interconnected complexes [36], [37] and the specificity of each type of elongation complex and of meromycolic modifications seems to be given by specific ternary interactions between condensing enzymes, dehydratase heterodimers and MA-Mts. These interactions are certainly crucial and their efficient targeting by inhibitors might be certainly detrimental for the survival of *Mtb*: it represent the targets of choice.

## Materials and Methods

### Strains and culture conditions

Plasmid constructions were done in the *Escherichia coli* K12 derivative Top10-F’ (Invitrogen) using classical cloning procedures and according to enzyme and product manufacturers (NEBiolabs, Fermentas, Promega). When needed, culture media (Luria-Broth (LB), or LB-Agar) were supplemented with kanamycin (50 µg/mL) or ampicillin (100 µg/mL).

The Y2H and Y3H recipient strains (Clontech) were *Saccharomyces cerevisiae* AH109 (MATa; trp1-901; leu2-3, 112; ura3-52; his3-200; gal4Δ; gal80Δ; LYS2::GAL1_UAS_-GAL1_TATA_-HIS3; GAL2_UAS_-GAL2_TATA_-ADE; URA3::MEL1_UAS_-MEL1_TATA_-lacZ) and MaV203 (MATα; leu2-3,112; trp1-901; his3Δ200; ade2-101; cyh2^R^; can1^R^; gal4Δ; gal80Δ; GAL1::*lacZ*; LYS2::GAL1_UAS_-GAL1_TATA_-HIS3; SPAL10::URA3 respectively. AH109 and MaV203 were cultured in YEP (BIO101) with 2% dextrose and 0.003% adenine. Selective plates were made with synthetic medium DOBA (BIO101) supplemented with the amino acids of the Complete Supplement Mixture (BIO101) lacking leucine and tryptophan (DOBA-LT) for AH109 or lacking methionine, leucine, and tryptophan (DOBA-LMT). The Y2H genetic tests were performed on DOBA-LT also devoid of histidine (DOBA-LTH), or adenine (DOBA-LTA), or both (DOBA-LTHA). The Y3H genetic tests were performed on DOBA-LTM also devoid of histidine (DOBA-LTMH), or uracile (DOBA-LTMU), or both (DOBA-LTMHU). When needed, the selective media were also supplemented with 1 mM L-Methionine.

### Construction of the Y2H and Y3H vector derivatives

The vectors pGBK-T7 and pGAD-T7 from the Matchmaker^™^ Two-hybrid system 3 (Clontech) allowed the downstream cloning of genes in phase with the coding sequence of the DNA-binding domain (BD) or of the activation domain (AD) of the yeast GAL4 transcription activator. In pGBK-T7, the BD coding sequence is followed by the sequence of an internal T7 promoter and the coding sequence of an eleven amino-acid epitope tag from the proto oncogene c-Myc, just upstream of the multiple cloning sites. After cloning of a gene of interest, the resulting protein will be a C-terminal fusion with the BD domain and the c-Myc epitope tag. The vector pGAD-T7 possesses the same genetic organization but it contains the coding sequence of the AD domain of GAL4, the sequence of a hemagglutinin (HA) tag, an ampicillin resistance gene and the yeast LEU2 coding sequence. The pBridge Y3H vector (Clontech) offers two cloning regions: a “pGBK-T7 like” cassette allowing the construction of BD fusions and a multiple cloning site downstream of a flexible pMet promoter (repressed by methionine). In addition, pBridge carries an ampicillin resistance gene and the yeast selection marker TRP1. The four *Mtb* MA-Mtf genes studied here; *cmaA1* (*rv3392c*), *cmaA2* (*rv0503c*), *pcaA* (*rv0470c*), and *umaA* (*rv0469*), together with the three dehydratase genes *hadA* (*rv0635*), *hadB* (*rv0636*), and *hadC* (*rv0637*) were all amplified by PCR from *Mtb* H37Rv chromosomal DNA using the *Pfu* DNA polymerase (Promega) with specific pairs of primers (Table S2) allowing their in phase cloning in the multiple cloning sites of pGAD-T7 and pGBK-T7. In addition, the three dehydratase genes were cloned as C-terminal fusion of the GAL4 BD domain in the pBridge vector. Each of the three resulting plasmid (pBridge::BD-HadA, pBridge::BD-HadB, pBridge::BD-HadC) was used to clone the remaining two others dehydratase genes under the pMet promore control. All the combinations of pairs of dehydratases were obtained. The control vectors (Clontech) encode for either a non-interacting protein (pGAD::*lam)* or a pair of strong interacting proteins *(*pGAD::*AgT* and pGBK::*p53).* They have been described in details in previous work [36], [37].

### Y2H and Y3H genetics analysis

AH109 has two main reporter genes (HIS3 and ADE2), under the control of two different GAL4 dependent promoters. The promoter driving the expression of HIS3 possesses a strong GAL4 Upstream Activating Sequences (UAS), and thus allows the detection of weak interactions. The promoter of ADE2 has a weak GAL4 UAS and thus allows only the detection of strong interactions. AH109 was transformed with each couple of pGAD-T7 and pGBK-T7 (or pBridge in Y3H) derivatives as described (Clontech). Co-transformants, containing two plasmids, were selected on DOBA-LT. As a first screen for protein-protein interactions and for each couple of plasmid tested, at least five individual co-transformants were streaked on DOBA-LT with replicate on DOBA-LTH, DOBA-LTA and DOBA-LTHA for AH109 derivatives or on DOBA-LT with replicate on DOBA-LTH, DOBA-LTU and DOBA-LTHU with or without L-Methionine (1 mM) for MaV203 derivatives and in the presence of the HIS3 inhibitor 3-amino-triazol (3-AT, 100 mM). Following this first screen, the validity of a given interaction was evaluated by plating dilutions (1.10^4^ and 1.10^3^) of saturated liquid cultures of an individual co-transformant on the eight types of selective plates. On each medium, the number of colony-forming unit (cfu) was reported to the number of cfu on DOBA-LT. Following the streak test and the plating assay, the scoring of a given interaction was done as follows: an interaction was scored as positive ( + ) when numerous individual colonies were visible on the streak assay and when more than 80% of the population had the appropriate phenotype on a given medium in the plating assay. It was scored as plus-minus ( +/- ) when only a few colonies were visible in the streak assay and when 50% to 80% of the population had the required phenotype in the plating assay. In all the other cases, the results were scored as negative ( - ).

### In vitro Co-immunoprecipitation

*In vitro* transcription/translation of the genes of interest was done with supercoiled DNA (1 µg) from pGAD-T7 or pGBK-T7 derivatives as matrix with the TnT^®^ Quick Coupled Transcription/Translation System (Promega). Reactions were performed in a final volume of 50 µL either in the presence of 0.4 µCi/µL of L-[^35^S]-methionine (1000 Ci/mmol; Amersham) or with cold methionine (40 µM) when unlabeled h-proteins were required. For Co-IP experiments, we used Dynabeads^®^ M-450 Goat anti-mouse IgG coated with monoclonal anti-HA antibodies (Sigma). The ratios of the proteins were adjusted to 1∶1 by evaluating the specific activity of each protein (in cpm per µL) and correcting by their differences in the number of methionine residues. Proteins were incubated with the coated beads (2 hours at 4°C) in 20 µL of 50 mM Tris (pH 7.4), 50 mM NaCl and 0.025% Tween 20. After extensive washing of the beads with 100 mM Tris (pH 7.4), 100 mM NaCl and 0.025% Tween 20, the reactions were boiled in the SDS PAGE loading buffer and fractionated on SDS PAGE followed by autoradiography and Phosphor-imaging (STORM-Applied Biosystems).

## Supporting Information

Table S1 Compilation of Y2H and Co-IP experiments between the FAS-II proteins and the MA-Mtfs.
(DOCX)

Table S2 Oligonucleotide sequences of PCR primers and cloning sites used for the construction of pGAD-T7, pGBK-T7 and pBridge derivatives.
(DOCX)
