# Supplementary material for: The Mycobacterium Tuberculosis FAS-II Dehydratases and Methyltransferases Define the Specificity of the Mycolic Acid Elongation Complexes
Source: PLoS One. 2011 Dec 22;6(12):e29564. doi: 10.1371/journal.pone.0029564 (PMC3245277; doi:10.1371/journal.pone.0029564)
Supplement: Table S1 — Compilation of Y2H and Co-IP experiments between the FAS-II proteins and the MA-Mtfs. (DOCX) [file pone.0029564.s001.docx]

**Table S1. Compilation of Y2H and co-IP experiments between the FAS-II proteins and the MA-Mtfs.**

| FAS-II proteins | MA-Mtfs | | | | | | | | | | | | | | | | | | | | | | | |
| --- | --- | --- | --- | --- | --- | --- | --- | --- | --- | --- | --- | --- | --- | --- | --- | --- | --- | --- | --- | --- | --- | --- | --- | --- |
|  | *cmaA1* | | | *cmaA2* | | | *umaA* | | | *pcaA* | | | *mmaA1 ^*^* | | | *mmaA2 ^*^* | | | *mmaA3 ^*^* | | | *mmaA4 ^*^* | | |
|  | Y2H1 | Y2H2 | IP | Y2H1 | Y2H2 | IP | Y2H1 | Y2H2 | IP | Y2H1 | Y2H2 | IP | Y2H1 | Y2H2 | IP | Y2H1 | Y2H2 | IP | Y2H1 | Y2H2 | IP | Y2H1 | Y2H2 | IP |
| *mabA* | - ^a^ | - | -^b^ | - | - | - | - | - | - | - | - | - | - | - | - | - | - | - | - | - | - | - | - | - |
| *inhA* | - | - | + | - | ± | + | - | - | + | - | - | + | - | - | + | - | - | + | - | - | + | - | - | + |
| *kasA* | + | + | + | + | + | + | + | + | + | + | + | + | + | - | + | + | + | + | + | + | + | + | + | + |
| *kasB* | ± | ± | + | ± | + | + | ± | ± | + | ± | ± | + | + | - | + | ± | + | + | + | + | + | + | + | + |
| *mtfabH* | - | ± | + | - | ± | + | + | ± | - | - | - | - | - | - | - | - | ± | - | - | ± | - | - | + | - |
| *mtfabD* | ± | + | + | ± | + | - | + | + | - | + | ± | - | + | + | - | + | + | - | ± | - | - | + | + | - |
| *pks13* | - | + | - | - | - | - | - | - | - | - | - | - | - | - | ± | - | - | ± | - | ± | ± | - | ± | ± |

1. Each sign indicates the growth on three selective media (DOBA-LTH, DOBA-LTA, and DOBA-LTHA) the rules of attribution of either +, ±, or – are given in Materials and Methods.
2. Each sign represents an evaluation of the co-IP experiments, the rules of attribution of either +, ±, or – are given in Materials and Methods or published previously [[36](#_ENREF_36)].

**Table S1. Compilation of Y2H and co-IP experiments between the FAS-II proteins and the MA-Mtfs.**

The results of Y2H experiments performed between AD-FAS-II protein fusions and BD-MA-Mtfs protein fusions are presented in the Y2H1 columns. In a similar fashion, the results of Y2H experiments in the reverse sense: between BD-FAS-II protein fusions and AD-MA-Mtfs protein fusions are presented in the Y2H2 columns. The results of Co-IP experiments are presented in the IP columns. The data concerning the MmaAs proteins (labeled with a star) correspond to previously published results [[36](#_ENREF_36)]. Similar weight was attributed to each type of experiment (Y2H1, Y2H3 or IP) in order to evaluate the final values presented on the Table 3.
