# Supplementary material for: The Mycobacterium Tuberculosis FAS-II Dehydratases and Methyltransferases Define the Specificity of the Mycolic Acid Elongation Complexes
Source: PLoS One. 2011 Dec 22;6(12):e29564. doi: 10.1371/journal.pone.0029564 (PMC3245277; doi:10.1371/journal.pone.0029564)
Supplement: Table S2 — Oligonucleotide sequences of PCR primers and cloning sites used for the construction of pGAD-T7, pGBK-T7 and pBridge derivatives. (DOCX) [file pone.0029564.s002.docx]

**Table S2. Oligonucleotide sequences of PCR primers and cloning sites used for the construction of pGAD-T7, pGBK-T7 and pBridge derivatives.**

| Gene | Site | PCR primers (5’ to 3’, left and right)^a^ | Target vectors |
| --- | --- | --- | --- |
| *hadA* (F) ^b^ | *Nde*1 | GGCCGCTCTCATATGGCGTTGAGCGCAGAC |  |
| *hadA* (R) | *Bam*H1 | CCGGCGTAGTTCACCGGATCCTGGCGGGTC | pGAD-T7, pGBK-T7, pBridge |
| *hadB* (F) | *Nde*1 | GAGAGGGATTTTCATATGGCGCTGCGTGAG |  |
| *hadB* (R) | *Eco*R1 | AAATCATCCCGCGGAATTCGGTCTTGAGCG | pGAD-T7, pGBK-T7, pBridge |
| *hadC* (F) | *Nde*1 | GAAGTTAGCGTAGCATATGGCGCTCAAGAC |  |
| *hadC* (R) | *Eco*R1 | AGCGCGCGTAATAGAATTCACTATAGGGCG | pGAD-T7, pGBK-T7, pBridge |
| *hadA* (F) | *Bgl*II | GGCCGCTCTAGATCTGCGTTGAGCGCAGAC |  |
| *hadA* (R) | *Bgl*II | CGTAGTTCACCAGATCTTGGCGGGTCAGCG | pBridge |
| *hadB* (F) | *Bgl*II | GAGAGGGATTTTAGATCTGCGCTGCGTGAG |  |
| *hadB* (R) | *Bgl*II | CCGCGGATATAGATCTTGAGCGCCATAAAC | pBridge |
| *hadC* (F) | *Bgl*II | GAAGTTAGCGTAGAGATCTGCGCTCAAGAC |  |
| *hadC* (R) | *Bgl*II | GATCGGTGCGGAGATCTTCGCTATTACGCC | pBridge |
| *cmaA1* (F) | *Nde1* | TACGCTATCCATATGCCCGACG |  |
| *cmaA1* (R) | *BamH1* | GAGATACTCAAGGATCCGACCGTCAAGAGC | pGAD-T7 and pGBK-T7 |
| *cmaA2* (F) | *Nde1* | AGGGTCCGTCATATGACGTCACAG |  |
| *cmaA2* (R) | *BamH1* | GGTGCGCGAGAGGATCCAGCAGAC | pGAD-T7 and pGBK-T7 |
| *umaA* (F) | *Nde1* | AGGTTCGGCCCATATGACTGAG |  |
| *umaA* (R) | *BamH1* | ACCCGGCGTGGGATCCGCAACC | pGAD-T7 and pGBK-T7 |
| *pcaA* (F) | *Nde1* | AGGCTCAATCCATATGTCCGTG |  |
| *pcaA* (R) | *BamH1* | CCAGCGCGCTGGATCCGGTTAC | pGAD-T7 and pGBK-T7 |

^a^ The restriction sites used for cloning in the target vectors are underlined

^b^ The orientation of the PCR primers with respect to the gene transcription is indicated into the brackets as forward (F) or reverse (R)
